# Supplementary material for: Remodeling of secretory lysosomes during education tunes functional potential in NK cells
Source: Nat Commun. 2019 Jan 31;10:514. doi: 10.1038/s41467-019-08384-x (PMC6355880; doi:10.1038/s41467-019-08384-x)
Supplement: Supplementary file 1 — Supplementary Information [file 41467_2019_8384_MOESM1_ESM.pdf]

Supplementary Information

Remodeling of Secretory Lysosomes During Education Tunes Functional Potential in NK Cells

Jodie P. Goodridge[1,2], Benedikt Jacobs[1,2], Michelle L. Saetersmoen[1,2], Dennis Clement[1,2], Quirin Hammer[3], Trevor Clancy[1,2], Ellen Skarpen[4], Andreas Brech[4], Johannes Landskron[1,5], Christian Grimm[6], Aline Pfefferle[3], Leonardo Meza-Zepeda[7,8], Susanne Lorenz[8], Merete Thune Wiiger[1,2], William E. Louch[9], Eivind Heggernes Ask[1,2], Lisa L. Liu[3], Vincent Yi Sheng Oei[1,2], Una Kjällquist[10], Sten Linnarsson[10], Sandip Patel[11], Kjetil Taskén[1, 2, 5], Harald Stenmark[4], Karl-Johan Malmberg[1,2,3]

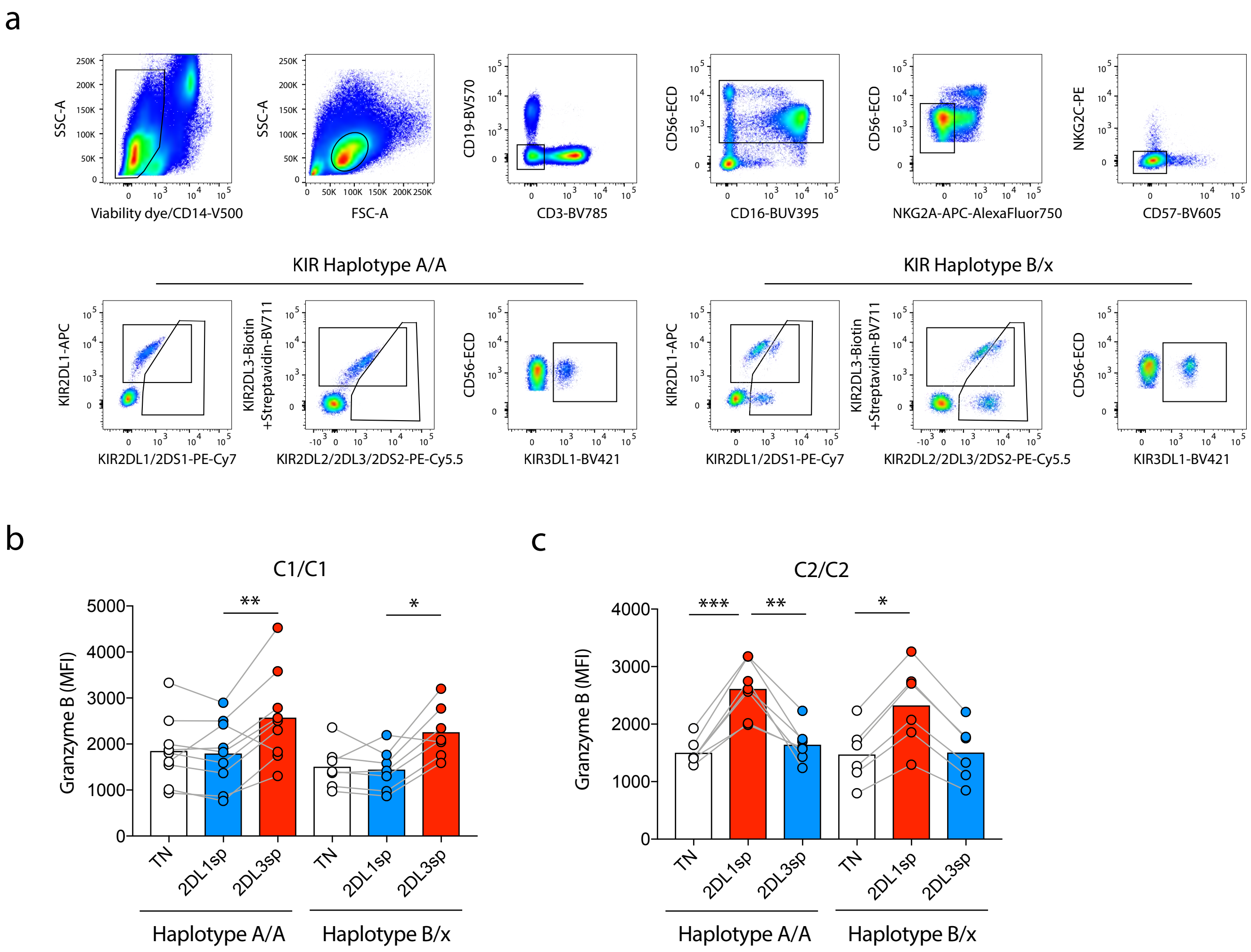

Supplementary Figure 1. Gating scheme and analysis of granzyme B expression in haplotype A/A and haplo-type B/x donors. (a) Example of gating scheme for analysis of expression of effector molecules in discrete NKG2A-CD57- NK cell subsets expressing single KIR. (b-c) Expression of granzyme B in NK cells triple negative (TN) for 2DL1, 2DL2/3, 3DL1 or expressing either of these KIR as their only KIR. Stratified analysis of donors with haplotype A/A and B/x in **(b)** HLA-C1/C1 and **(c)** HLA-C2/C2 donors. Red and blue colors indicate expression of self and non-self KIR, respectively. Friedman's test followed by Dunn's multiple comparison test were performed in panels b and c.**; p < 0.001; **p < 0.01; and *p < 0.05.

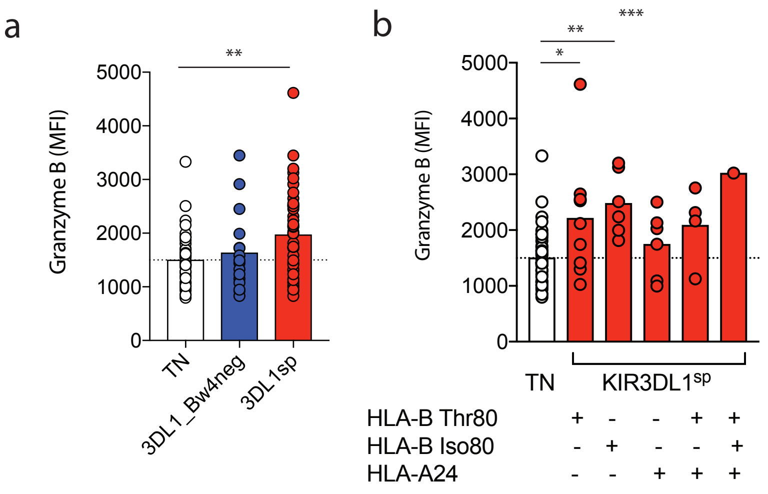

Supplementary Figure 2. Granzyme B expression stratified based on KIR3DL1 and HLA-Bw4 ligands. **(a)** Expression of granzyme B in the indicated NKG2A-CD57- NK cell subset from Bw4+ (n=26) and Bw4- donors (n=16). TN=triple negative for 2DL1, 2DL3 and 3DL1. 3DL1sp=3DL1 single-positive. **(b)** Substratification showing expression of granzyme B in 3DL1sp NK cells based on the Bw4 ligands Bw4^{Thr80} (n=9), Bw4^{Iso80} (n=6), Bw4^{A24} (n=6), Bw4$^{Thre80+A24}$ (n=4) and Bw4$^{Iso80+A24}$ (n=1) were two rare to be analysed separately. One-way ANOVAs followed by Tukeys' multiple comparison test were performed in panels a and b. ***; $p < 0.001$; **$p < 0.01$; and *$p < 0.05$.

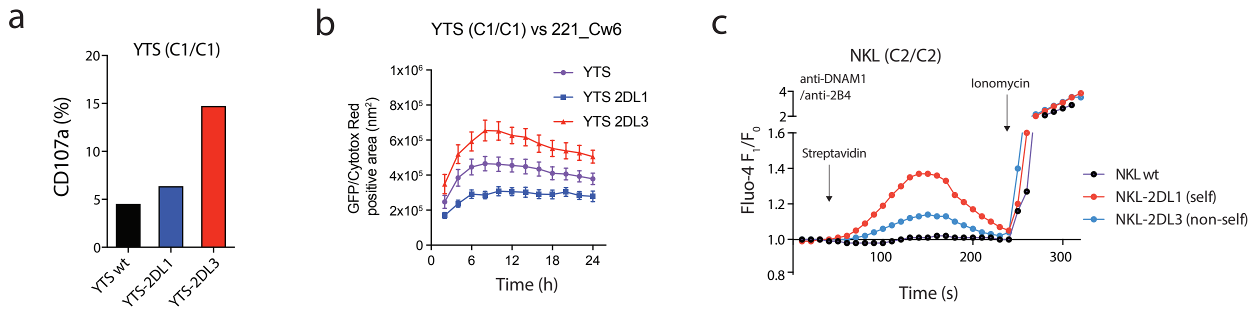

Supplementary Figure 3. Functional responses in KIR-transduced NK cell lines. (a) Degranulation in YTS cells transduced to express self (2DL3) and non-self (2DL1) KIR. **(b)** Long-term cyto-toxicity assay in the Incucyte showing killing (GFP+Cytotox Red+ area) of 221.Cw6.GFP+ cells by the KIR engineered YTS lines E:T ratio 1:1. Bars represent SD of triplicates. **(c)** Ca^{2+}-flux in NKL cells transduced with self (2DL1) and non-self (2DL3) KIR in response to crosslinking of DNAM-1/2B4. Graphs in panels a-c show one representative of two experiments.

a

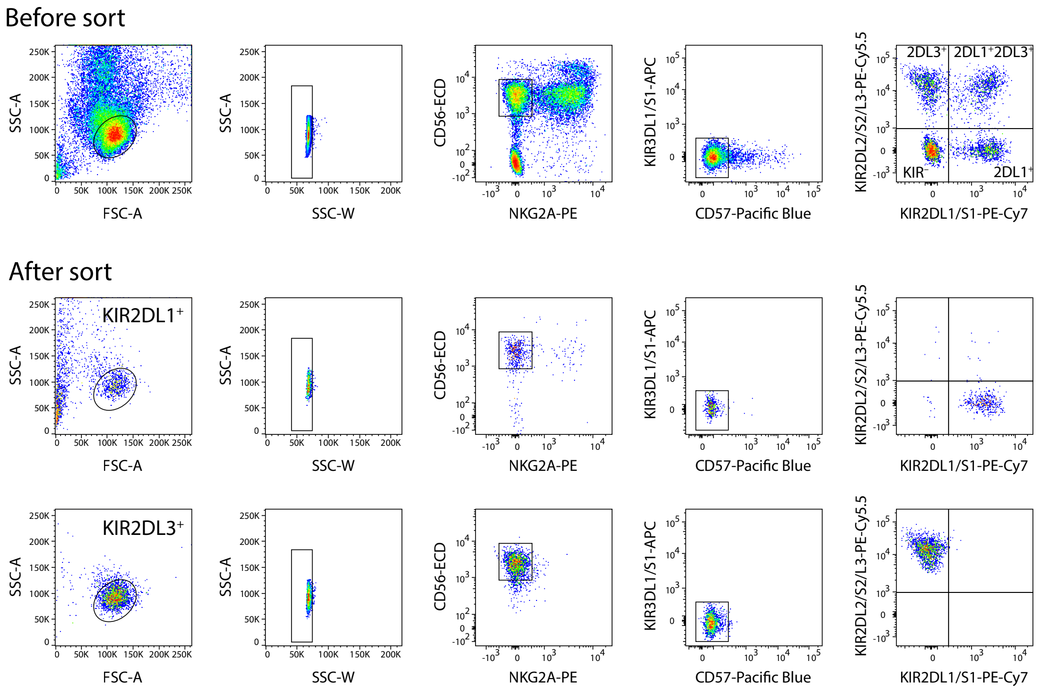

b

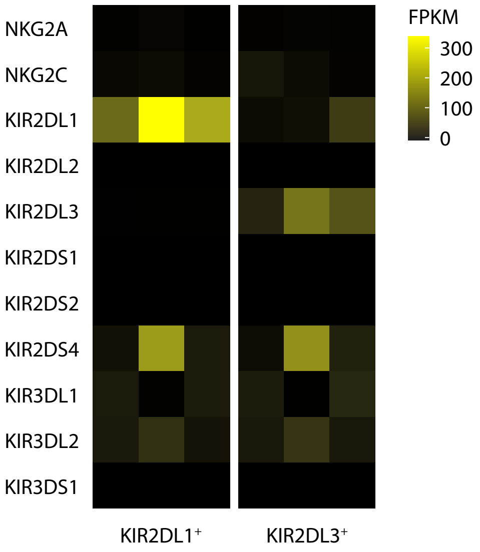

Supplementary Figure 4 .Purity of sorted subsets. (a) Representative example of NK cell phenotypes before and after flowcytometry-based sort from an haplotype A C1/C1 donor. **(b)** RNA-Seq of sorted 2DL1$^+$ (left) or 2DL3$^+$ (right) NKG2A$^-$NKG2C$^-$CD57$^-$CD56^{dim} NK cell subsets. Data are from three representative donors. FPKM; fragments per kilobase of exon per million reads mapped.

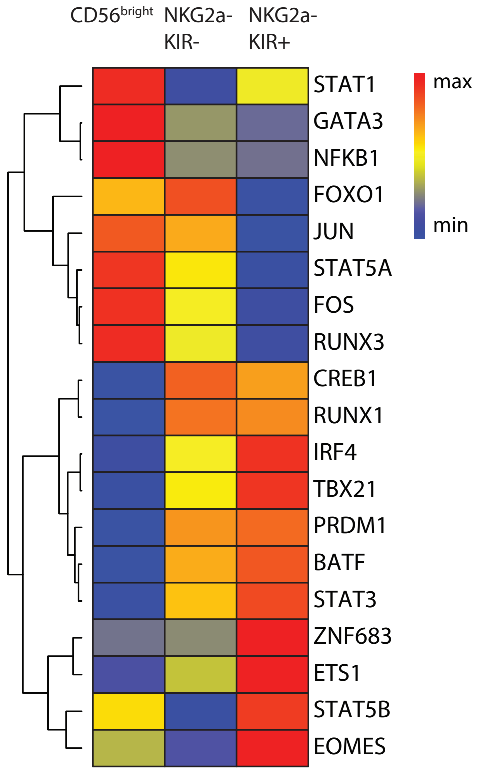

Supplementary Figure 5. Transcriptional regulation of NK cell differentiation. Global RNA-Seq of sorted CD56^{bright}, NKG2A^{-}panKIR^{-} and NKG2A^{-}panKIR^{+} CD56^{dim} NK cell subsets. Shown are genes involved in the regulation of cytokine responsiveness and transcription of effector molecules, including gran-zyme B. Data are from one representative donor out of three independent donors.

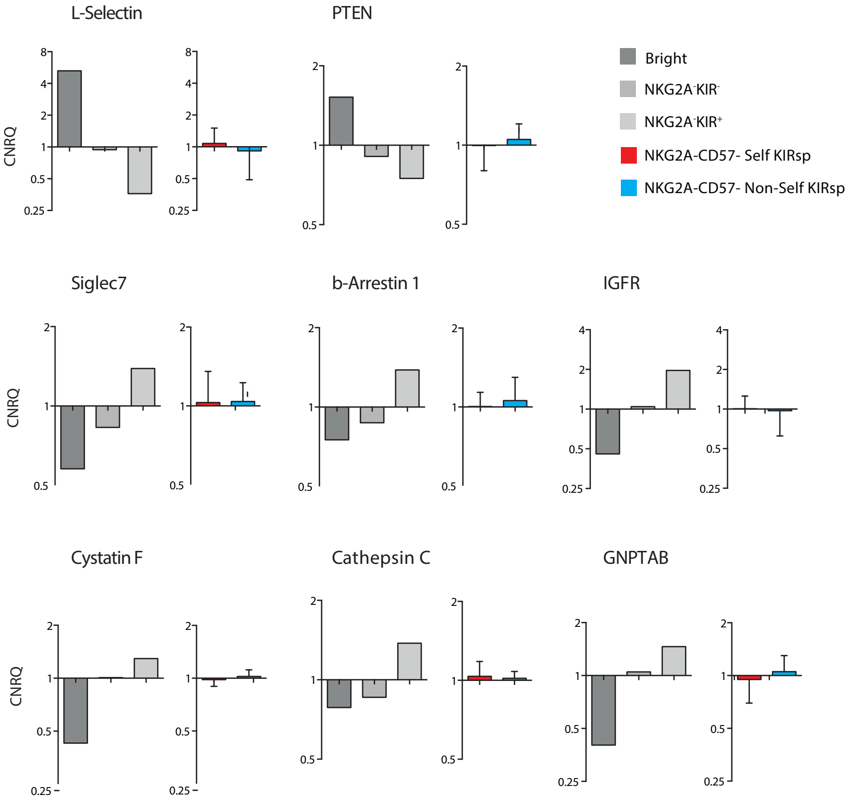

Supplementary Figure 6. Transcriptional dissociation of differentiation and education. Quantitative PCR of mRNA of the indicated markers in sorted CD56^{bright}, NKG2A^{-}panKIR^{-} and NKG2A^{-}panKIR^{+} CD56^{dim} NK cell subsets (left, 4 pooled samples) as well as in sorted NKG2A^{-} CD57^{-} CD56^{dim} NK cells expressing self or non-self KIR (right, n=5 paired samples). Markers showing a positive relationship with differentiation were selected for comparison with education subsets. Whiskers show 5th to 95th percentile.

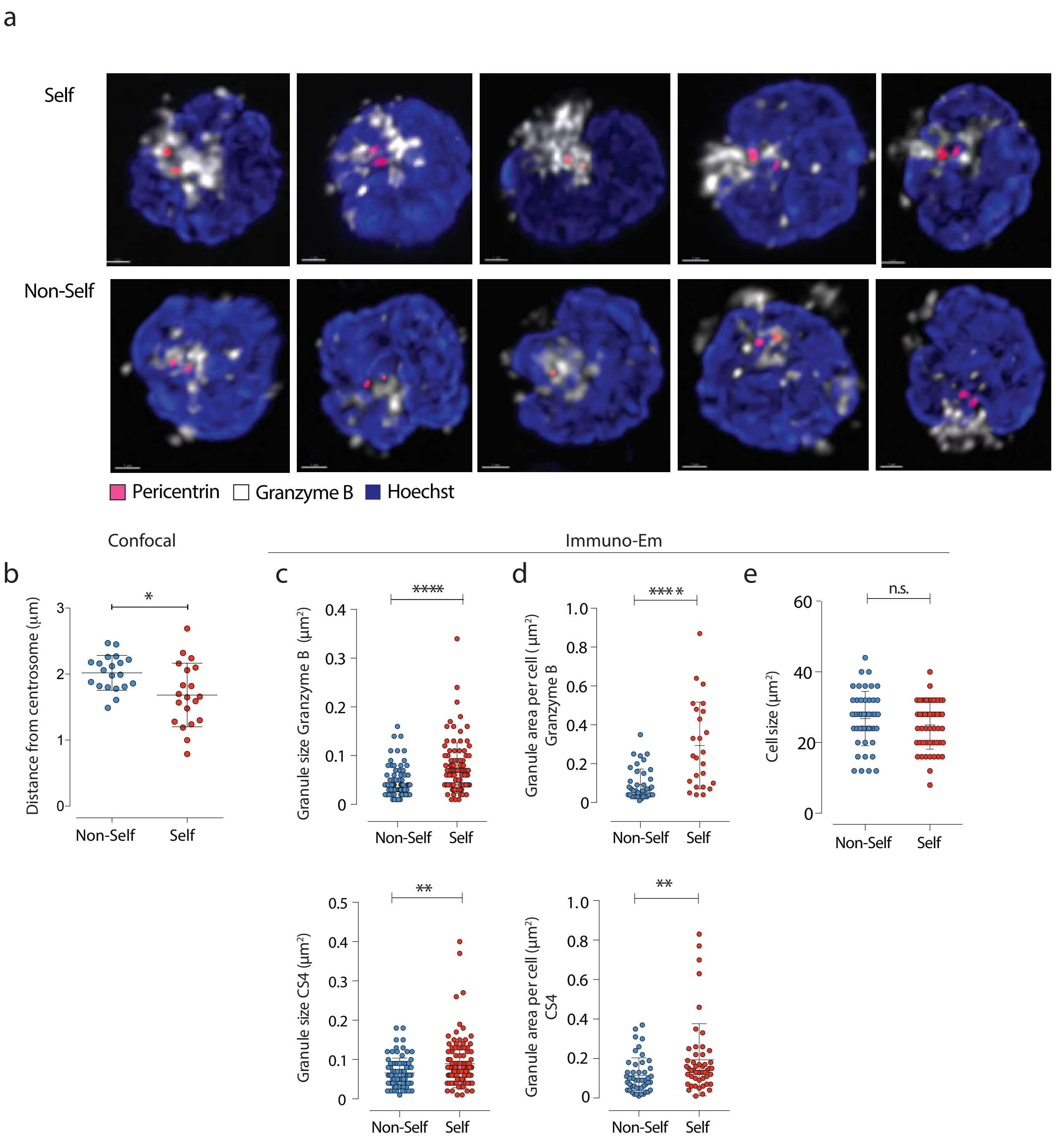

Supplementary Figure 7. Expression of self-KIR is associated with accumulation of large granzyme B dense secre-tory lysosomes. (a) Confocal Z-stacks showing Pericentrin (PCNT) in red and granzyme B staining in white in sorted CD56dim NKG2A-CD57- NK cells expressing Self (top) or Non-Self KIR (bottom). A panel of representative phenotypes acquired from one representative donor out of 5 is shown. **(b)** Average distance from the centrosome of the brightest lysosmal structures (top 25%) in confocal microscopy, n=20 cells per condition. **(c)** Granule size as determined by using grid overlay of electron dense structures staining posi-tive for granzyme B (upper panel) and Chondroitin Sulphate-4 (CS4) (lower panel). **(d)** Granule area per cell as determined by taking total calculated granule area per cell for structures staining positive for gran-zyme B (upper panel) and CS-4 (lower panel). **(e)** Comparison of cell size between Self (n=50) and Non-Self (n=49) KIR+ NK cells. Whiskers show 5th to 95th percentile. Bars show the median. Non-parametric Mann-Whitney tests were performed in panels b-e. ****$p < 0.0001$**; $p < 0.01$; and *$p < 0.05$

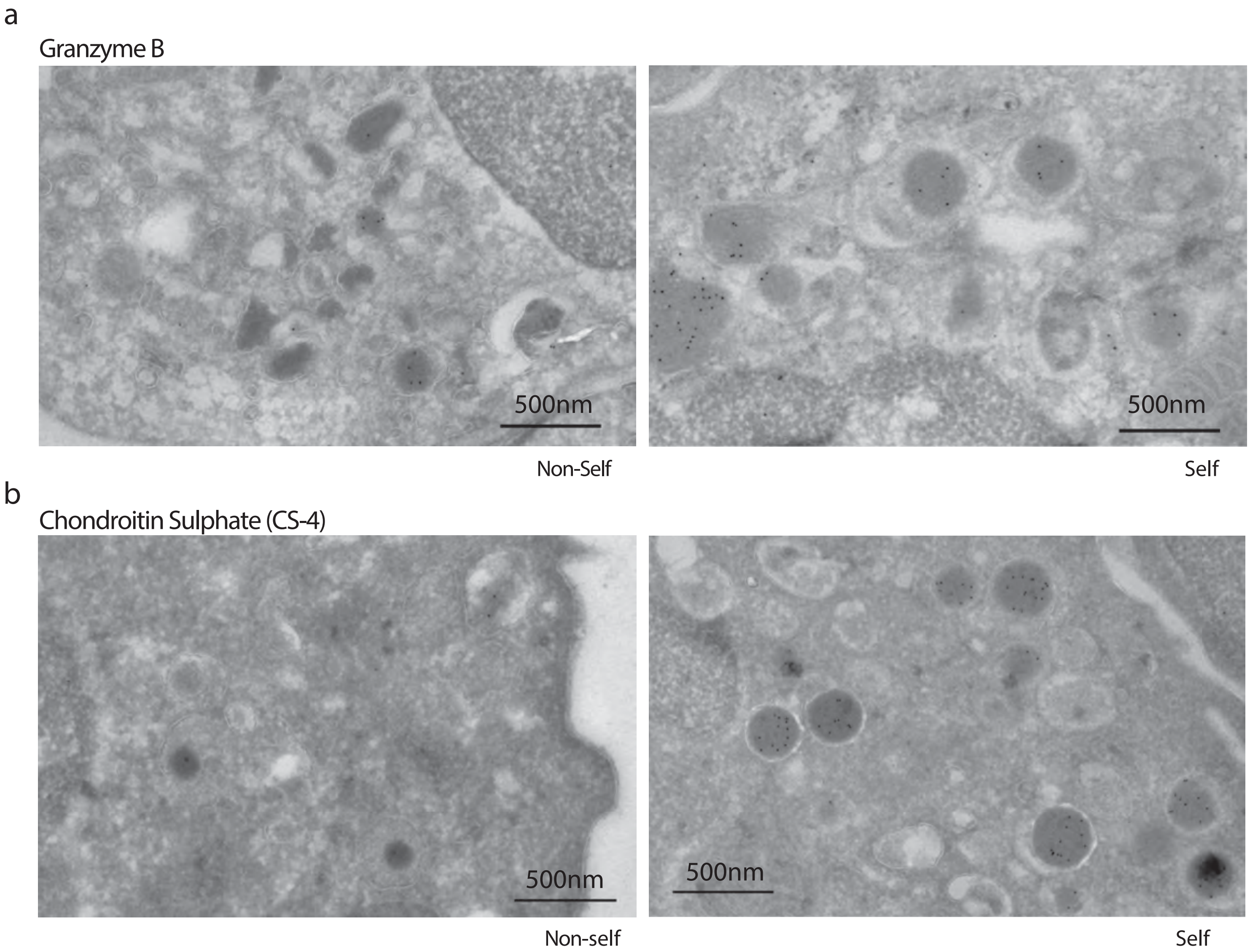

Supplementary Figure 8. Immuno-EM of sorted self/nonself KIR^{+}NKG2A^{-}CD57^{-} CD56^{dim}NK cells. Representative immuno-EM sections showing staining with gold-particle coated anti-granzyme B **(a)** and chondroitin Sulphate-4 (CS-4) **(b)** of sorted CD56^{dim} NKG2A^{-}CD57^{-} NK cells expressing non-self (left) or self KIR (right). Immuno-EM images are from sections of representative cells from n=5 donors and n=5 experiments.

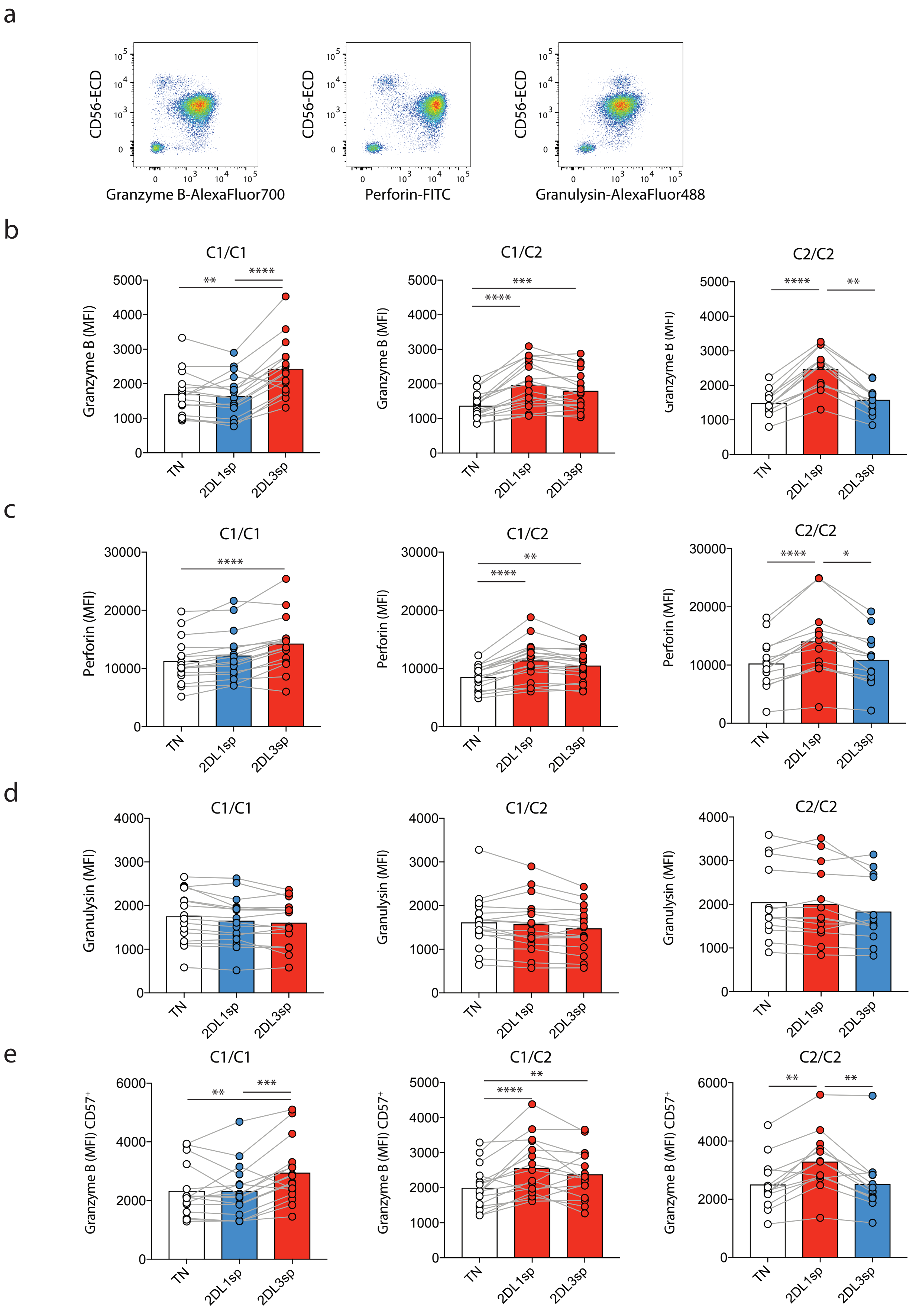

Supplementary Figure 9. NK cell education through self-KIR is associated with accumulation of granzyme B and perforin but not granulysin. (a) Representative examples showing expression of granzyme B, perforin and granulysin in $CD56^{bright}$ and $CD56^{dim}$ NK cells. Expression of **(b)** granzyme B **(c)** perforin and **(d)** granulysin in $NKG2A^{-}CD57^{-}$ NK cells expressing the indicated KIR in C1/C1 (n=16), C1/C2 (n=18) and C2/C2 (n=13) donors. KIR2DL1 single-positive (2DL1sp). (e) Expression of granzyme B in $NKG2A^{-}CD57^{+}$ NK cells expressing the indicated KIR in C1/C1 (n=16), C1/C2 (n=18) and C2/C2 (n=13) donors. Friedman's test followed by Dunn's multiple comparison test were performed in panels b-e. In panel d, there was no statistical accumulation of granulysin in any of the KIR+ subsets. ****p < 0.0001***; p < 0.001; **; p < 0.01and *p < 0.05.

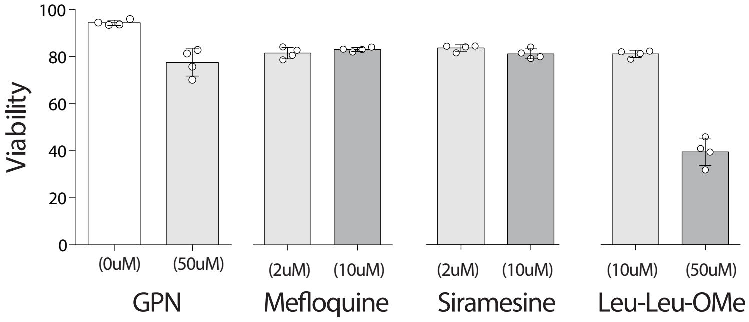

Supplementary Figure 10. Viability of NK cells exposed to lysosomotropic compounds. Viability was determined by titration of each of the indicated compounds during 4 hours by monitoring incorporation of a live/dead marker for cell viability using flow cytometry. Shown are the mean of 4 replicates. Whiskers show standard deviation.

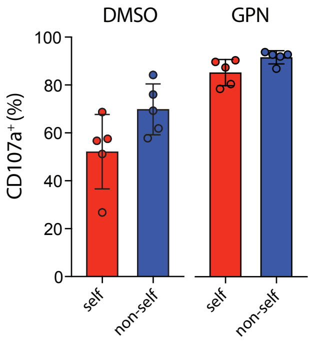

Supplementary Figure 11 Compromising lysosomal activity increases the functional response PMA/Ionomycin stimulation. Resting NK cells were treated with PMA (1ng/mL) and Iono-mycin (0.5 μM) for 4 hours in the presence of DMSO (control) or GPN at 50μM (n=5). Whiskers show standard deviation.

Supplementary Table 1

Effector Transcription	mTOR Signaling			
NFATC1	AKT3	ATP6V1H	SKP2	STRADA
RUNX1	RRAGB	PDPK1	SLC3A2	ATP6V1F
IKAROS	LAMTOR5	CAB39	SOS1	EIF4E2
JUN	NPRL2	ATP6V1A	SOS2	ATP6V1G1
FOS	RRAGA	ATP6V1B1	BRAF	FNIP1
NFKB1	FZD10	ATP6V1B2	STK11	TTI1
MITF	CHUK	ATP6V1C1	TNF	DEPDC5
IRF4	ATP6V1G3	ATP6V1E1	TNFRSF1A	ULK2
BATF	SLC38A9	PIK3CA	TSC1	TELO2
EOMES	DVL1	PIK3CB	TSC2	TBC1D7
TBX21	DVL2	PIK3CD	WNT1	-LOC100130357
PRDM1	DVL3	PIK3CG	WNT2	
STAT1	EIF4B	PIK3R1	WNT3	
STAT3	EIF4E	PIK3R2	WNT5A	
STAT5A	EIF4EBP1	ATP6V1G2	WNT6	
STAT5B	FLCN	WNT4	WNT7A	
ETS1	AKT1	MIOS	WNT7B	
RUNX3	AKT2	DDIT4	WNT8A	
CREB1	RNF152	LAMTOR1	WNT8B	
GATA3	LPIN1	STRADB	WNT10B	
FOXO1	PIK3R5	PRR5	WNT11	
	ATP6V1C2	PRKAA1	WNT2B	
	MTOR	PRKAA2	WNT9A	
	RICTOR	PRKCA	WNT9B	
	EIF4E1B	PRKCB	FZD5	
	FZD2	PRKCG	MAPKAP1	
	ULK3	MAPK1	WDR59	
	RPS6KA6	MAPK3	FZD3	
	GRB2	MAP2K1	WNT10A	
	GRB10	MAP2K2	WNT5B	
	LAMTOR2	PTEN	NPRL3	
	GSK3B	RPTOR	SLC7A5	
	HRAS	FNIP2	CAB39L	
	IGF1	RRAGD	SEH1L	
	IGF1R	RAF1	FZD1	
	IKBKB	RHEB	FZD4	
	INS	RPS6	FZD6	
	INSR	RPS6KA1	FZD7	
	IRS1	RPS6KA2	FZD8	
	KRAS	RPS6KA3	FZD9	
	RHOA	RPS6KB1	SESN2	
	LAMTOR4	RPS6KB2	ULK1	
	LRP6	CLIP1	WDR24	
	LRP5	SEC13	AKT1S1	
	NRAS	RRAGC	PIK3R3	
	TBC1D7	MLST8	LAMTOR3	
	ATP6V1D	SGK1	WNT3A	
	WNT16	DEPTOR	ATP6V1E2	

Lysosomal Biogenesis			
A2M	*F2R*	*RAB26*	*TMED10*
ABCA1	*F2RL3*	*RAB27A*	*TMED2*
ABCA12	*F5*	*RAB4A*	*TOR1A*
ABCC4	*F8*	*RAB4B*	*VAMP1*
ACTB	*FGA*	*REST*	*VAMP2*
ACTN1	*FGB*	*SELP*	*VAMP7*
ACTN2	*FGG*	*SERPINA1*	*VAMP8*
ACTN4	*FN1*	*SERPINA5*	*VEGFA*
ADAM8	*GAL*	*SERPINE1*	*VPS33B*
ALDOA	*GAS6*	*SERPINF2*	*VWF*
AP1G1	*GHRL*	*SFTPD*	*ZP3*
APOA1	*HGF*	*SLC11A1*	
APP	*HPS4*	*SLC2A4*	
AZU1	*HRG*	*SNAP23*	
BLOC1S1	*HSPD1*	*SNAPIN*	
BLOC1S2	*IGF1*	*SNCA*	
BLOC1S3	*IGF2*	*SNX19*	
BRCA2	*INS*	*SNX19*	
CAPZB	*ITGA2B*	*SPACA1*	
CASC5	*ITGB3*	*SPACA3*	
CCDC64	*KIT*	*SPARC*	
CD36	*KLK7*	*SPESP1*	
CD63	*LRGUK*	*SPINK5*	
CD9	*LYST*	*SRGN*	
CHGA	*MPO*	*SRI*	
CLCN3	*MYO5A*	*STX3*	
CLU	*OLFM4*	*STX4*	
CPE	*PAFAH1B1*	*STX7*	
CTSG	*PDGFA*	*STXBP1*	
CTSH	*PDGFB*	*STXBP3*	
CXADR	*PECAM1*	*TEKT3*	
CYBA	*PLG*	*TF*	
DENND4C	*PRKCA*	*TGFB1*	
DTNBP1	*PTPRN*	*TGFB2*	
EGF	*RAB10*	*TGFB3*	
ELANE	*RAB11B*	*THBS1*	
EXOC3	*RAB13*	*TIMP1*	

Genes involved in effector transcription, mTOR signaling and lysosomal biogenesis.

Supplementary Table 2

Gene Symbol	Alias	Refseq #	Official Full Name	RT2 Catalog Number
PPC	PPC	SA_00103	Positive PCR Control	PPX63339
HGDC	HIGX1A	SA_00105	Human Genomic DNA Contamination	PPH65835
RTC	RTC	SA_00104	Reverse Transcription Control	PPX63340
18SrRNA		X03205	Human 18S ribosomal RNA	PPH05666
B2M	-	NM_004048	Beta-2-microglobulin	PPH01094
ARRB1	ARB1/ARR1	NM_004041	Arrestin, beta 1	PPH02341
GZMB	CCPI/CGL-1/CGL1/CSP-B/CSPB/CTLA1/CTSGL1/HLP/SECT	NM_004131	Granzyme B (granzyme 2, cytotoxic T-lymphocyte-associated serine esterase 1)	PPH02594
CST7	CMAP	NM_003650	Cystatin F (leukocystatin)	PPH05560
SELL	CD62L/LAM1/LECAM1/LEU8/LNHR/LSEL/LYAM1/PLNHR/TQ1	NM_000655	Selectin L	PPH00677
CTSC	CPPI/DPP-I/DPP1/DPPI/HMS/JP/JPD/PALS/PDON1/PLS	NM_001814	Cathepsin C	PPH06129
GNPTAB	GNPTA/ICD	NM_024312	N-acetylglucosamine-1-phosphate transferase, alpha and beta subunits	PPH19573
IGF2R	CD222/CIMPR/M6P-R/MPR1/MPRI	NM_000876	Insulin-like growth factor 2 receptor	PPH00351
PTEN	10q23del/BZS/CWS1/DEC/GLM2/MHAM/MMAC1/PTEN1/TEP1	NM_000314	Phosphatase and tensin homolog	PPH00327
TBX21	T-PET/T-bet/TBET/TBLYM	NM_013351	T-box 21	PPH00396
SIGLEC7	AIRM1/CD328/CDw328/D-siglec/QA79/SIGLEC-7/SIGLEC19P/SIGLECP2/p75/p75/AIRM1	NM_014385	Sialic acid binding Ig-like lectin 7	PPH15594
MCOLN1	TRP-ML1	NM_020533	Mucolipin 1	PPH19799A
